# Practitioners’ ability to remotely develop understanding for personalised care and support planning: a thematic analysis of multiple data sources from the feasibility phase of the Dementia Personalised Care Team (D-PACT) intervention

**DOI:** 10.1177/14713012231185281

**Published:** 2023-06-24

**Authors:** Hannah Wheat, Sarah Griffiths, Alex Gude, Lauren Weston, Cath Quinn, Sarah Morgan-Trimmer, Tomasina M Oh, Crispin Musicha, Leanne Greene, Mike Clark, Sarah Rybczynska-Bunt, Richard Byng

**Affiliations:** Community and Primary Care Research Group, 6633 University of Plymouth, Plymouth, UK; Centre for Ageing Population Studies, Research Department of Primary Care and Population Health, 4919 University College London, London, UK; Community and Primary Care Research Group, 6633 University of Plymouth, Plymouth, UK; Department of Health and Community Sciences, Faculty of Health and Life Sciences, 3286 University of Exeter, Exeter, UK; Community and Primary Care Research Group, 6633 University of Plymouth, Plymouth, UK; Medical Statistics, Faculty of Health, 6633 University of Plymouth, Plymouth, UK; Department of Rehabilitation, Aged and Extended Care, Flinders University, Adelaide, SA, Australia; Care Policy and Evaluation Centre, 4905 London School of Economics and Political Science, London, UK; Community and Primary Care Research Group, 6633 University of Plymouth, Plymouth, UK

**Keywords:** remote interaction, dementia, personalised care and support, communication, thematic analysis

## Abstract

Practitioner understanding of patients’ preferences, wishes and needs is essential for personalised health care i.e., focusing on ‘what matters’ to people based on their individual life situation. To develop such an understanding, dementia practitioners need to use communication practices that help people share their experiences, preferences, and priorities. Following the COVID-19 pandemic, dementia support is likely to continue to be delivered both remotely and in-person. This study analysed multiple sources of qualitative data to examine the views of practitioners, people living with dementia and carers, and researchers on how an understanding of what matters to people living with dementia can be developed remotely via telephone and video call. Access to environmental stimuli, the remote use of visual tools, peoples’ tendency to downplay or omit details about their troubles and carers’ ability to disclose privately were interpreted, through thematic analysis, to be factors affecting how practitioners sought to develop understanding remotely. Cumulatively, findings show that while remote support created unique challenges to practitioners’ ability to develop understanding for personalised care, practitioners developed adaptive strategies to overcome some of these challenges. Further research should examine how, when and for whom these adapted practices for remote personalised care work, informing the development of evidence-based guidance and training on how practitioners can remotely develop the understanding required for personalised care.

## Introduction

Over 850,000 people in the United Kingdom (UK) live with dementia and this is predicted to increase to around 1.6 million by 2040 (Wittenberg et al., 2019) – increasing in turn, the demand on the social and health care service to provide tailored, timely and effective dementia support.

The successful delivery of personalised care is key to services meeting the support needs of people with dementia in the UK, providing more independence, choice and control over support received (Alzheimer’s Society, 2022). Consequently, it is NHS England’s (2020a) mission to ensure that every person living with dementia has a personalised support plan, based on “what matters to them, paying attention to their needs and wider well-being” (p.7).

Practitioner understanding of patients’/clients’ preferences, wishes and needs, within the context of their unique life situations, is essential for holistic ‘personalised care and support planning’ (PCSP). Despite cognitive and communication challenges, people with dementia often have the desire and ability to enable their support network to understand their individual priorities and values (Daly et al., 2017). Consequently, practitioners working with people living with dementia and their carers need to be able to communicate in ways that help people share who they are (Hobson, 2012).

Following the onset of the severe acute respiratory syndrome coronavirus 2 (SARS-CoV-2) pandemic, UK primary care services began to interact with patients, including those with dementia, via telephone and video consultations instead of face-to-face consultations (Majeed et al., 2020; Park, Elliott, Berlin et al., 2020). It is highly likely that some aspects of health care (including dementia care) will continue to be provided remotely, as well as in-person (NHS England, 2020b; NHS England and NHS Improvement, 2021; Scottish Government, 2022).

Trials of remote consultations, with people with dementia, using video technology have demonstrated that this is a feasible format for providing ongoing care, if there was a preceding face to face visit explaining the remote consultation or the provision of additional technology and support (Adams et al., 2020; Choi & DiNitto, 2013; Friemel, 2016). However, Tuijt et al (2021) highlight that digital exclusion among older populations, especially those with a lower income and lower (eHealth) literacy is high compared with other age groups (Friemel, 2016) and found that while remote consultations could be effective for people with dementia, they could benefit from being more focused around new physical health needs. Additionally, remote consultations could also benefit from people with dementia using communication strategies (e.g., checklists), as a means of replacing non-verbal prompts available in face-to-face consultations, to help them remember and describe problems.

Other literature exploring remote communication in dementia support interactions has focused on group support (e.g. Dowson and Schneider., 2021; Perkins et al., 2022); exercise programmes (e.g. Di Lorito et al., 2021); and/or reports only from the view of staff delivering remote support (e.g. Madden, Rose, & Crystal, 2021; Wheatley et al., 2021) or carers, without the perspectives of people with dementia (e.g. Gonzalez-Fraile et al., 2021; Baruah et al., 2021). Despite the importance of the subject, there has been a lack of evidence about patients’ views (NIHR- Applied Research Collaboration West, 2021).

Notably, despite the importance placed on delivering personalised care to people with dementia, there has been no study specifically examining the extent to which practitioners can remotely develop an understanding of ‘what matters’ to people required for PCSP.

Consequently, this study sought to examine multiple experiences and perceptions of remotely delivered care (that aims to provide PCSP), from practitioners, independent observers (researchers) and, most importantly, from people with dementia and informal carers by utilising an existing data set from the D-PACT (Dementia - PersonAlised Care Team) project, in which the aim was to deliver personalised care to people living with dementia and informal carers through a dementia support worker.

## Method

*Context - the nature of the support provided and received within the D-PACT study:* The D-PACT intervention is a coaching-based intervention for people with dementia **and** informal carers, in which the practitioners’ understanding of ‘what matters’ to people is central to them engaging in care planning and shared decision making. This intervention was developed during a two-year feasibility phase. The intervention is delivered by dementia support workers (DSWs) embedded in General Practice (GP) surgeries.

D-PACT DSWs aim to develop personalised understanding of the people they support (both people with dementia and their carers, if the person with dementia has one and they wish to also take part) by taking time to get to know people before engaging in coaching-based steps, including using visual (written or pictorial) tools depicting potential areas that people may wish to focus on (e.g., memory and thinking, emotions, physical health, people and relationships, activities, and planning ahead for finances, healthcare and legal issues). These tools were informed by existing evidence on life domains relevant and meaningful for both people with dementia and their carers (e.g., Bielsten et al., 2018; Dening & Aldridge, 2019). The aim is to support people with dementia and informal carers to articulate what matters to them, identify links between areas of concern, and co-develop a ‘shared plan’ to address matters of importance (maintain what is working well/change what is working less well). This coaching approach is highly flexible. In cases where there is an ongoing crisis, a more proactive/problem solving approach is adopted.

The DSW collaborates with other professionals to enhance the personalised care the whole team provides to individuals (hence the name of the intervention/project - Dementia PersonAlised Care team), and offers to be a single point of contact. The role involves providing information, tailored to the person’s unique needs, about support available across service sectors. This may involve problem solving barriers to access such as transport and confidence issues. DSWs can also support transitions, for instance from the care of one service to another, or to care home living. People with dementia and carers are offered the opportunity to meet with the DSW together and/or separately. This was deemed important as it would: 1), create opportunities for people with dementia and carers’ understanding of each other’s needs/preferences to be enhanced through triadic (support) interactions, 2), show respect of individuals’ preferences for the carer/person with dementia to remain with them during support meetings; 3), acknowledge how carers could support the interaction and 4) importantly, allow for one to one discussion of sensitive topics. Joint decisions on who the DSW would meet with and when were made during initial support meetings and then revisited during the length of the support. DSWs could suggest topics were continued in one to one settings, if they felt a current topic discussion topic may be causing distress for one of the parties or that one party would benefit from the opportunity to talk freely without fear of upsetting the other. Separate meetings were treated confidentially unless the person with dementia/carer agreed that information could be shared in a triadic meeting. Prior to COVID-19, support meetings were intended to be face-to face either at the clients’ home or at their GP surgery.

D-PACT DSWs had only been delivering support (as part of the feasibility phase of the project) for 6 months before the COVID-19 pandemic social restrictions were put in place – resulting in DSWs having to rapidly adapt to delivering support remotely over the telephone or via a video call.

### Recruitment and selection of participants

The study reported on here draws from data collected from participants who took part in the feasibility stage of the D-PACT project, which has now been completed.

*Recruitment of people with dementia and carers:* People with dementia and informal carers were recruited from 10 GP surgeries during September 2019 - March 2021 (extended recruitment phase due to COVID-19) in Southwest (SW) and Northwest (NW) England. The inclusion criterion for a person with dementia to be enrolled onto the D-PACT feasibility study was that they had a clinical diagnosis of dementia identified through pre-defined SNOMED codes (SNOMED is a global systematised suite of clinical vocabulary and coding used in electronic care records). Inclusion criterion for carers was that they were the main, unpaid carer for the person with dementia. Please see Table 1 for a summary of the recruitment figures for the feasibility study.

**Table 1.** Recruitment figures for D-PACT feasibility study.

	People with dementia	Carers
Recruited	54	52
Excluded after consent – too high on the Telephone Interview for Cognitive States (TICS: Brandt et al., 1988)	2	N/A
Number recruited from practices allocated to intervention arm (i.e., received support from the D-PACT support worker)	44^a^	44^a^
Number recruited from practices allocated to control arm (i.e., received treatment/care as usual)	9	9

^a^Includes one dyad which did not receive support – were not allocated to DSW.

A purposeful sub-sample of the intervention cohort of people with dementia and carers dyads was created for this study (n = 16 dyads – 16 carers and 16 people with dementia) based on whether they received support remotely (see Table 2 for the study sample’s demographics). Based on preference, practicalities and support needs, interviews were carried out with these 32 participants individually or as dyads. Interviews were undertaken as part of the larger study, exploring experiences of personalised care and support.

**Table 2.** The demographics of the study sample.

	Age (median)	Sex	Nationality	First/native language	Living situation	Carer employment
Person with dementia (n = 16)	77 (Interquartile range 72.0, 85.2)	Male = 7 (44%) Female = 9 (56%)	British = 15 (94%) Caribbean = 1 (6.2%)	English = 16 (100%)	Lives alone = 2 (12%) Lives with main carer = 10 (62%) Lives with main carer and others = 4 (25%)	/
Carer (n = 16)	70 (Interquartile range 58, 74)	Male = 8 (50%) Female = 8 (50%)	British = 16 (100%)	English = 16 (100%)	/	Working full time = 2 (12%) Working part time = 3 (19%) Retired = 10 (62%) Long-term sick or disabled = 1 (6.2%)

*Recruitment of practitioners:* At the time of this study, 5 practitioners were employed on D-PACT (2 supervisors and 3 DSWs), funded through the project grant. These practitioners were NHS employees who were recruited through advertisements in the NHS Trusts at the two sites - seconded onto the study for the duration of the project. Consequently, DSWs continued to be employed by the NHS and line managed by a NHS staff member, while also receiving supervision from the recruited D-PACT DSW supervisor. DSWs ranged from NHS band 3 – 4, and supervisors were employed at band 6. All had completed mandatory NHS training relevant to their level. Job requirements were that DSWs would have some experience of working with people living with dementia (experiences of relevant experience include working on an in-patient ward caring for people with advanced dementia, holding a different dementia support worker role and conducting dementia research) and supervisors would have extensive experience of providing dementia care and support, case management and multi-disciplinary team working (relevant experience included roles as a senior mental health and admiral nurse). Bespoke training, supported by a practitioner manual, was developed delivered to DSWs and supervisors by the research team. This included interactive face-to-face sessions involving an expert in coaching within the dementia field.

All practitioners involved in this phase of the study took part in interviews that contributed to these analyses.

*Ethics:* A favourable opinion was received from the South Central - Berkshire Research Ethics Committee for the study. Informed consent was either written or verbally provided. Participants (people with dementia and carers) and practitioners, were asked for their consent to provide a range of qualitative data (including semi-structured interviews, recordings of support meetings and practitioner diaries) for use as data within analyses, and as anonymised examples within publications, conference presentations and future practitioner training. People with dementia and carers were given the choice about involvement in these methods of data collection and were assured that they could withdraw from these aspects of data collection at any time, without affecting their ongoing receipt of the intervention.

***Capacity and Consent:*** People with dementia who lacked capacity to consent were still able to participate in the feasibility study, if they assented, and if a suitable consultee provided consent on their behalf. Once enrolled onto the study, both carers and people with dementia were approached for interview after they had received support and if they had indicated they were open to being interviewed in their original consent form. If they were still amenable to being interviewed, they were then asked for their consent to be interviewed and for the recording and transcript of their interview to be used as data. Full details of our consent and capacity process and reasons why people with dementia were excluded have been reported elsewhere (see Griffiths et al., 2022).

### Data collection

Table 3 outlines the data collected for this study. We conducted 17 semi structured interviews with people with dementia and carers, using an interview guide that sought to explore intervention recipients’ views and perceptions on the personalised support they received. Exploring to what extent they developed a relationship with the DSW, whether they felt understood and in control of the support and whether the support focused on what mattered to them. The semi-structured format of the interview schedule included questions relating to the tenets of personalised care, but also gave researchers opportunities to ask probing questions exploring individuals’ experiences and perceptions and generate new insights (DeJonckheere & Vaughn, 2019). Researchers were committed to enabling the interaction to shape the interview’s direction, rather than rigidly sticking to an interview schedule, thereby enabling the collection of meaningful data from a small number of participants (DeJonckheere & Vaughn, 2019). All interview participants had received support remotely and questions within the schedule focused on their experiences of receiving support in this way. Interviews were conducted remotely, via telephone or a video call, due to covid-19 social distancing rules prohibiting researcher-participant face to face contact. Potential limitations of this are explored in the discussion section. The interviews typically lasted around 60 minutes.

**Table 3.** Data collected for study.

	Type of data collected		
Participant(s) data collected from	Semi-structured interview	Non-participant observations	Practitioner diary reflections
Person with dementia and carer dyad	8		
Carer only	7		
Person with dementia only	2		
Person with dementia, carer and DSW		6^a^	
Carer with DSW		5^a^	
Practitioner – DSW	3		50 diary entries
Practitioner – supervisor	2		
Total	22	11^b^	50 diary entries^c^

^a^One dyad and one carer provided 2 observations, in all other instance participants only provided one recorded observation.

^b^Six observations were only of audio recorded support sessions, 5 observations were of video and audio recorded support sessions.

^c^Two of the DSWs (one in SW and one in NW) opted to keep a reflective diary. The extent of diary entries made by each DSW was disparate (43 entries by the SW DSW and 7 by the NW DSW).

DSWs and their supervisors were also interviewed (n = 5). Interviews explored the same issues relating to personalised care as the interviews with people with dementia/carers, but from a delivery perspective. These semi-structured interviews lasted between 60-90 minutes and also had to be conducted remotely.

The decision on when to stop collecting interview data was informed by the number of people interviewed from the overall recruited sample and a review of whether we had sufficient information to inform the next stage of the D-PACT project (the evaluation) – which involved refinement of the intervention and its underpinning programme theory.

Data collected for the non-participant observations (Liu & Maitlis, 2010; Spradley, 1980) of remote support meetings (between the D-PACT DSW, person with dementia and/or carer) were naturalistic, as researchers were never present during the recording, and all sessions (n = 11) would have taken place irrespective of the recording occurring. Unstructured observations (Mulhall, 2003) were undertaken, serving to overcome challenges with recall and participant bias and provide a more “nuanced and dynamic” insight into how people with dementia, carers and DSWs interacted during support sessions – which could not be provided through the other sources of data collection (Liu & Maitlis, 2010). Researchers engaged in multiple viewings of recordings and made fieldnotes, guided by our design of the intervention and our theory on how it should work, as well as how practitioners were delivering it in practice and how people with dementia and carers were responding.

Practitioners’ reflective diaries (n = 50 diary entries) were intended to be relatively unstructured and kept as a means of documenting the “flow of public and private events that [were] significant to the diarist’ (Plummer, 1983, p. 17), enabling the immediacy of experiences/feelings to be captured (Fouad, 2021; Symon & Cassell, 1998) without being constrained by an imposed structure. However, practitioners were asked to specifically focus on their experiences delivering their support remotely, in addition to reflecting on what worked well/less well in specific cases, their own development, and the impact of support on their own emotions/thoughts/wellbeing.

### Data analysis

Reflexive thematic analysis was used to examine experiences and perceptions relating to the remote development of practitioner understanding of what matters to the people they support. This methodological approach enabled us to identify patterns across a large, mixed qualitative data set and for our coding process to be as “unstructured and organic” as possible (Braun & Clarke, 2021); with codes inductively created and advanced we increasingly developed our understanding of the data. We followed the six phases of reflexive thematic analysis outlined by Braun and Clarke (2021): familiarisation; coding; generating initial themes; reviewing and developing themes; refining, defining and naming themes and writing up.

Initially, HW (the main analyst) and SG familiarised themselves with the data set by reading transcripts of the interviews, the practitioners’ reflective logs and fieldnotes, and by watching the recorded support meetings. Initial observations were noted during this phase and, due to the prevalence of observations regarding the development of understanding across the data sets, this was selected as a focal point for the subsequent coding and analysis. Data relating to the development of understanding was coded inductively and managed within NVivo 12. Coding involved a line by line reading of each data source – looking for segments within the data that potentially offered meaningful insights, relevant to the study aims. Identified sections were given an “analytically-meaningful” (Braun & Clarke, 2022) code label that adequately described the insight provided. There were two rounds of coding, during which codes labels were refined. The majority of coding was undertaken by HW, but authors SG, AG, LG and TMO also coded data and attended coding discussions, so that the coding process could be informed by varied interpretations and opportunities for co-reflection. Potential themes were identified by printing off NVivo reports of data coded to each individual code, then examining and tracking back and forth between coded data and researcher memos– and noting where coded data appeared to relate to a shared or different meaning. HW then sought to interpret the core concept underpinning coded data that continued to appear to be related. Potential themes were reviewed with authors SG, AG and CQ, revised, and then finalised through group discussion. Further revision was undertaken after feedback from a researcher, external to the team.

*Reflexivity:* We acknowledged and addressed our influence on the study by attending to how data collection and analysis were influenced by our own characteristics, knowledge and experiences (personal reflexivity) as well as our methodological decision-making (epistemological reflexivity), (Lazard & McAvoy, 2020). We did this by 1) making short notes of thoughts we had during data collection, and coding, 2) writing longer reflective diary entries after was completed analysis and 3) discussing our reflections at analysis meetings. Examples of these reflections included: a tendency by researchers to refer to face-to-face engagement as preferrable to remote contact in some of the interview questions (the impact of this was checked and did not appear to directly influence the response); the suitability of using NVivo to generate themes (HW opted to move out of NVivo to interpret potential themes, so that a more latent, rather than semantic reading of the data could be achieved) and the influence of our involvement in developing the intervention and its impact on what we observed within the data (shared understanding is a core principle underpinning the intervention – by focusing on this from the onset we may have overlooked other areas of interest, important to the participants, within the data).

*Self-appraisal:* The qualitative critical appraisal skills programme (CASP) tool, was also used to self-appraise the quality of the study (CASP, 2018). The appraisal questions were all responded to positively except Question 5, ‘Was data collected in a way that addressed the research question?’ This required discussion, as data collection was primarily focused on the aims of the overarching project, rather than this specific study. A limitation related to this issue is detailed in the discussion.

## Results

Themes are grouped under two headings relating to the type of practitioner understanding, which literature and policy have underscored as essential for PCSP: (1) an understanding of what matters to people being supported and (2) a holistic understanding of a person’s situation; their strengths and needs. We present data contributing to the development of each theme, collected from people with dementia and informal carers (interviews), practitioners (interviews and reflective diaries) and the researchers (observations).

### Heading One: Practitioners developing an understanding of what matters to people they support

#### Theme One: Access to environmental stimuli to generate and support ‘what matters’ conversations

DSWs reported that not being in the person’s home limited their ability to initiate conversations that helped them get to know what was important to individuals. This was due to the lack of (if contact via telephone) or limited (if contact via video call) environmental stimuli, such as photographs, pets, a view of the garden, and seeing the person themselves, to help identify meaningful and important conversation topics and observe (non-verbal) reactions to those topics.


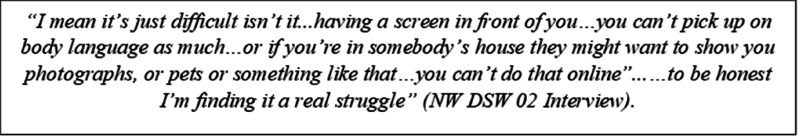


DSWs adapted to this in both phone and video calls by creating prompt notes on possible topics of importance (informed by clients’ verbal cues) for further discussion in subsequent meetings. DSWs reported that this practice helped remote conversations continue to flow naturally. While this is a practice that could help in both in-person and remote meetings, DSWs reported that the remote nature of the meeting made it easier for them to make, and later refer to, notes without it disrupting the conversation – as people with dementia /carers were less aware (or at the very least their attention was less drawn to the fact) that the use of notes was occurring.


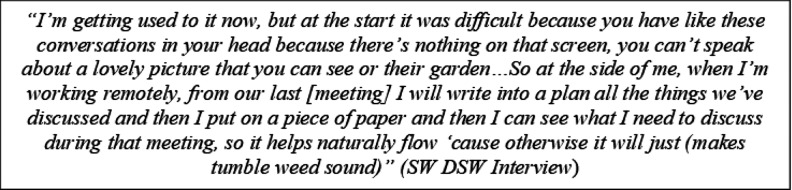


People with dementia and carers highlighted how meaningful it was to them when the DSW picked up on a topic of interest to them and then explored it further - demonstrating the value of DSW using strategies, such as crafting a plan of things to talk about, to help develop rapport and support conversational flow.


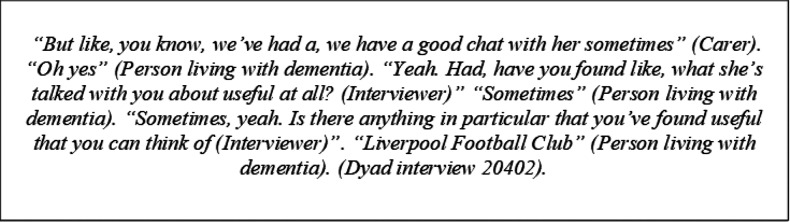


Researchers’ observation notes of recorded remote support meetings reinforced DSW perspectives, as they noted that difficulties in conversational flow at the start of the support meetings that potentially arose from DSWs starting conversations through “how are you doing” style questions, rather than through reference to environmental stimuli that may represent an interest of the individual(s) supported.


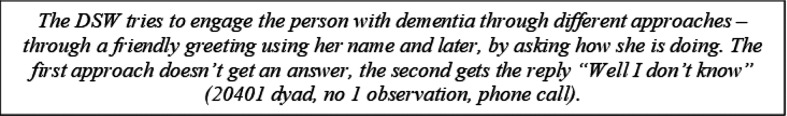


People with dementia and carers reported both an awareness of how limited environmental stimuli may affect the DSWs’ understanding of them, and how they addressed this. They reported that, when interacting via a video call, they would bring photos of interest to the computer screen, to show and explain to the DSW. People with dementia and carers’ reports on this experience highlighted how important it was to them that the DSW could build an understanding of who they were and what mattered to them, and how much they appreciated somebody showing genuine interest.


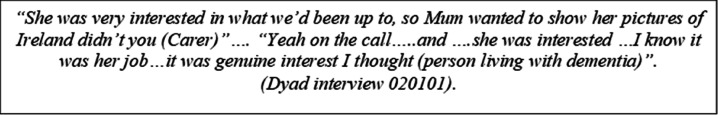


Researcher observation notes of recorded support meetings captured details on how sharing and talking about photos often led to visible signs of enjoyment for people with dementia and carers (evidenced through smiling and maintained engagement in the discussion). Such sharing also prompted reminiscing by the person with dementia of events that were important to them. This encouraged engagement of people with dementia and supported the DSW to build a picture of the person with dementia as an individual.

#### Theme Two: The remote use of visual tools to aid the development and checking of practitioners’ understanding of what matters

D-PACT DSWs are provided with two written, visual intervention tools. The ‘What matters to you’ tool (informed by the SHERPA model of shared decision-making - Jack et al., 2018; Swancutt et al., 2021) was designed to serve various purposes: 1), to highlight the range of areas within peoples’ lives that they could discuss and receive support on from the D-PACT DSW; b), facilitate the sharing of what matters to people with dementia and carers with the D-PACT DSW, and c), to aid discussion on how the biopsychosocial areas of peoples’ lives deemed important for discussion may be interlinked (see Appendix A). The ’Plan of action’ was designed to be a co-developed (by DSW and clients) document detailing what actions had been agreed upon, which could then be reviewed as revised as appropriate as time went on (See Appendix B).

Practitioners reported difficulties sharing these tools, especially when using their NHS video call software, which did not enable document sharing. They also found it difficult to explain the purpose of the tool and how to use it, without them being able to physically demonstrate how to use it to with the people they were supporting. The hoped-for benefits of using visual tools were not being realised due to the remote support delivery approach.


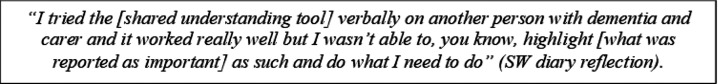


One approach taken by a DSW to overcome the difficulties sharing the ‘Plan of action’ tool remotely was to complete the tool on their own, then check whether their holistic understanding of the situation aligned with the understanding of the person with dementia/carer. However, interview data from their supervisor (after this approach had led to some interactional difficulties between the DSW and a dyad she supported) highlighted the importance of the tool being used collaboratively.


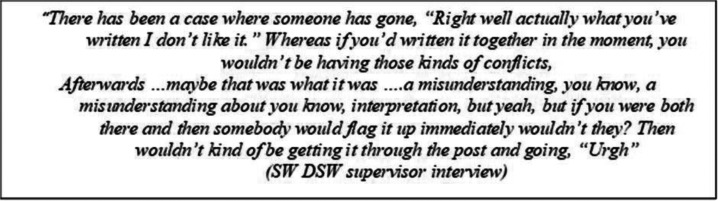


Such experiences suggest that, without practitioner understandings being co-established and checked for during real time collaborative tool completion (which is inhibited in online interactions), there is a risk that practitioners will inadvertently re-introduce a power differential in support, by presuming they understand when they do not. There is also the risk that people supported remotely will misunderstand the purpose of the tool and/or feel un-heard - thwarting the aims of PCSP. However, observational data of the plan being used in-person is needed before remote/in-person comparisons can be confidently made.

### Heading Two: A holistic understanding of a person’s situation; their strengths and needs

#### Theme Three: DSWS’ reliance on peoples’ verbal accounts of their situation to detect and understand changes in care needs

Part of the D-PACT DSW role is to monitor for changes in the health and wellbeing of the people they support and discuss those changes with them. With people’s permission the DSW can report changes in circumstances and need in their electronic medical records (e.g. medication seems not to be working well). It is the hope that early, shared, identification of emerging difficulties or deteriorations can enable a timely response, potentially averting an escalation of need or crisis situation (e.g., a new condition developing, or a fall). Identification and discussion of positive changes can help reaffirm peoples’ sense of control and empowerment, especially when they, themselves, have acted to bring about those changes, and this can elucidate how they might make beneficial changes to other areas of their lives.

Within this data set, DSWs reported concerns that they were unable to identify and understand *increased* needs for care when interacting remotely, because they did not have access to visual information to help detect change.


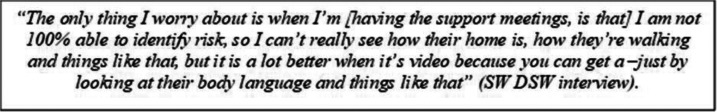


Researchers’ observation notes highlighted how the visual window into people’s lives provided by video calls was often very limited.


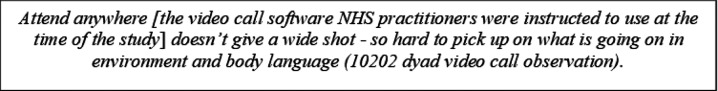


DSWs voiced concerns about whether they could rely solely on people’s verbal accounts of their situation to provide them with enough understanding of what their needs were.


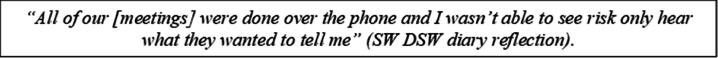


These uncertainties built on the DSWs’ perception that the people they supported would often either downplay how difficult they were finding things or would omit details about what they were finding difficult when asked questions about how they were.

In some data, the cause of this behaviour could be interpreted as the remote nature of the interaction. Other data supported an interpretation that some people already had a tendency for self-restraint, in terms of how much they spoke about their problems.

#### Environmental factors influencing self-restraint in trouble talk

Both practitioners and people with dementia and carers commented on how the lack of physical proximity impacted the ability for them to connect with one another and the impact this may have had on how comfortable people felt to disclose information about something that was concerning them or causing them difficulty. In the quote below, the supervisor suggests that a cup of tea at the person’s home may help facilitate the type of discussion where people are more likely to disclose “what’s truly worrying them”– suggesting that a lack of physical proximity and/or the opportunity to engage in rapport building physical behaviours (such as the ritual of making and receiving a drink) may limit the extent of shared understanding that can be achieved remotely.


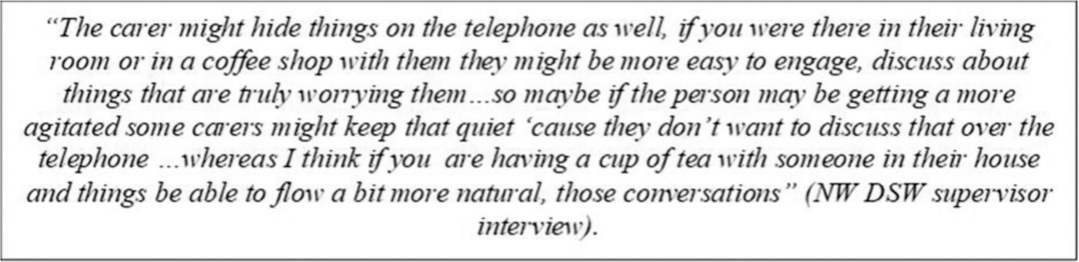


Supporting this suggestion were reports from people with dementia and carers that lamented the loss of connection that physical proximity and the engagement in face-to-face rapport building behaviours can provide.


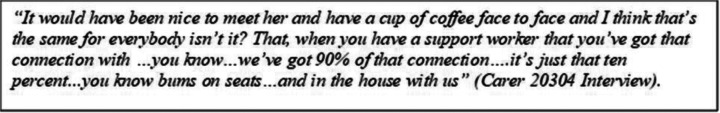


#### A tendency to downplay troubles

Researcher observation notes reported that people seemed to habitually downplay the extent of their difficulty when DSWs asked questions about how they were. They also questioned the extent to which DSWs could gain an accurate understanding of the situation of the person with dementia/carers, if they could only rely on what was said (i.e. without environmental stimuli or nonverbal behaviour) – especially if they were supporting a person with dementia on their own and there was no carer to provide further detail about their situation.


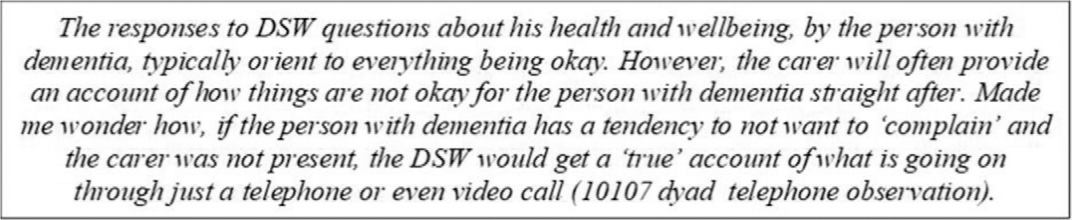


These types of observations were also made by DSWs, who highlighted how important it was not to place too much weight on initial responses to their ‘how are you’ questions as people tended to give generic, no-problem oriented responses, no matter what their situation was.

#### Interactional strategies to advance understanding when people exercised self-restrain during trouble talk

Within the data, certain types of interactional behaviour from DSWs appeared to address the potential reasons for why people may have not been forthcoming with details about their troubles.

- *DSW behaviour that sought to forge a personal connection in a remote setting*

DSW behaviour that helped forge a connection, and consequently encourage engagement and disclosure, included the display of warm and friendly gestures through non-verbal behaviour and self-disclosure.


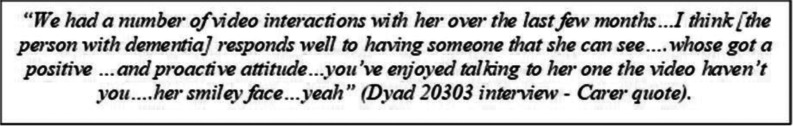



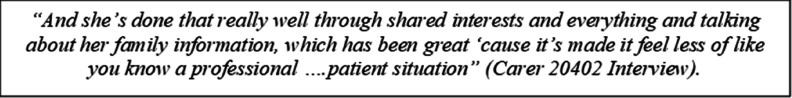


While the ability to display warm and friendly non-verbal behaviour was limited to video calls, D-PACT practitioner training resources encouraged DSWs to send photos of themselves, prior to their first meeting with people they support. Diary reflections from the DSWs included references to how this step was especially useful for people with dementia and carers who were only interacting with them via a telephone.

Connection-forging self-disclosure appeared to be facilitated by DSWs first identifying topics they had in common with the people they were supporting. People with dementia and carers especially found it beneficial when the DSW was local and could engage in discussions about their area. This may have been viewed as especially helpful due to the age gap between the DSW and person with dementia/carer potentially limiting the availability of other shared topics of interest. However, observation notes captured instances of DSWs skilfully initiating and maintaining various topics of discussions with people with dementia and carers. By sharing details of their own lives and engaging in a discussion about those subjects, the DSW could be viewed as conveying to the people with dementia/carer that they are someone who they enjoy engaging with and are someone who has something valuable to say – making use of verbal social rituals we all use to build connection with others in an interactional situation where physical actions to build rapport (e.g., share a cup of tea) are not possible.

- *DSW behaviour that attended to, and sought peoples’, verbal accounts of their situation in more nuanced ways*

One DSW spoke of the value of remaining vigilant for verbal clues that implicitly suggested things were not okay, within no-problem oriented talk. In the quote below, the DSW gives examples of the types of clues she would look out for, which include the use of double negatives (e.g. “not that bad”- suggesting there was some level of ‘bad’) or the implicit expression of a challenge by referring to positive behaviour they have to enact (“e.g. “I’m coping”- suggesting there was a challenge they had to cope with).


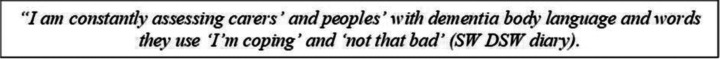


Another DSW spoke of how they would listen to *how* the person spoke about their situation as well as to what was said and would pay attention to whether they seemed engaged, and open to support, to help decipher how accurate the account of their situation was.


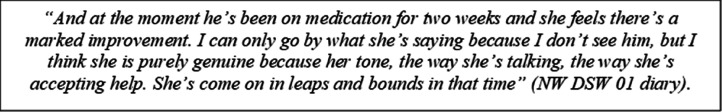


Researchers observed the use of certain styles of questioning, by DSWs, to reduce the likelihood of them receiving a ‘no problem’ oriented response. DSW intervention training emphasised the importance of using open questions during support meetings and these type of questions were observably used by DSWs to initiate topic discussion throughout support meetings. However, observation notes reported that DSWs were switching to closed questions as a means of accessing targeted information and of overcoming no-problem oriented or generic responses to open questions such as ‘how are you’ (e.g., a generic response would be “I’m fine’). These questions were sometimes designed to elicit just a yes or no response to an enquiry for specific information e.g. “Are you eating well?” The use of such questions did lead to the interaction becoming more ‘institutional’ (less coaching oriented/client driven) for a short amount of time – with the DSW leading a sequence of talk, which quickly moved through a succession of questions and answer responses. However, these closed question sequences did appear to elicit information about things that were not going so well in their lives and which could constitute risk of a substantial negative event (e.g. a fall or other accident, rapid deterioration, or a new condition developing, such as a urinary tract infection leading to delirium etc).


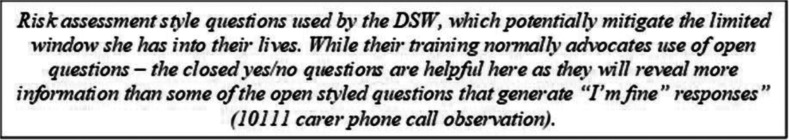


DSWs noted, themselves, the value of including a time frame in their questions about how the person with dementia/carers currently felt “e.g. how are you feeling at this specific time’ rather than asking for an overall (with no time specific framing) impression of how they were doing, e.g., ‘how are you feeling’. They reported that this could mitigate receiving a generic, no problem-oriented response.

In addition, they spoke of how targeted questions, during video calls, could be used specifically to explore the visual observations they *could* make about possible changes in specific symptoms/states, which helped them to expand the window they had into their current situation.


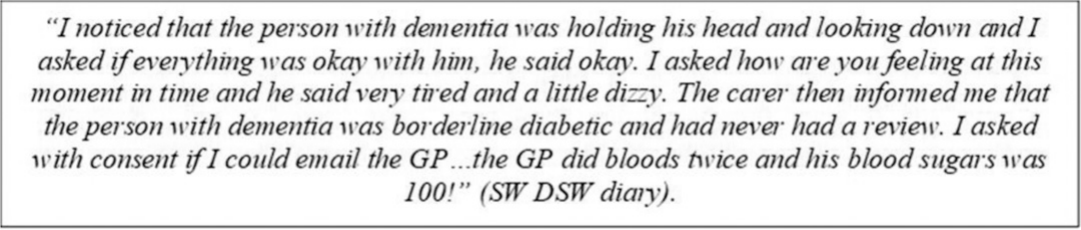


People with dementia and carers also expressed an awareness that DSWs were asking the right sort of questions to get to know them and build up a picture of what their current situation is, so that they could best support.


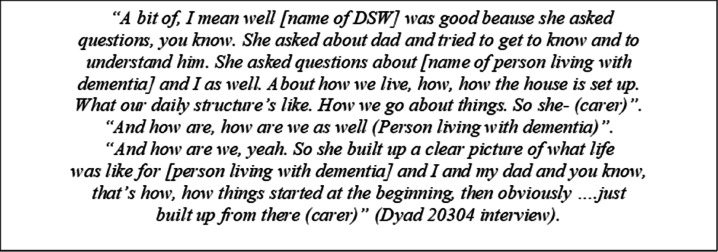


While interacting via video calls (compared to phone calls) substantially aided DSWs’ ability to pick up on problems that were not verbally expressed by the people they supported, observation notes indicated that DSWs were able to pick up on potential changes in situation for the person with dementia/carer by attending to non-lexical, emotive displays that suggested everything may not be okay, which were available in both video and phone calls, and then using these to inform gently inquisitive questions.


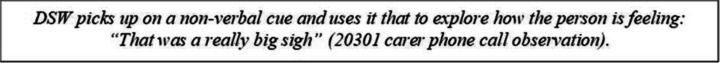


The importance of DSWs using such strategies, when problem talk was not forthcoming, to build not only understandings of need, but also to inform subsequent suggestions of how problem areas could be addressed is demonstrated in the account below. Within this account, a person with dementia and carer describe how a lack of information about their lives led a DSW to initially make inappropriate suggestions about what they may try to do to improve their situation. In their account, they propose that these suggestions were made because the DSW could not see the extent of the mobility difficulties for the person with dementia.


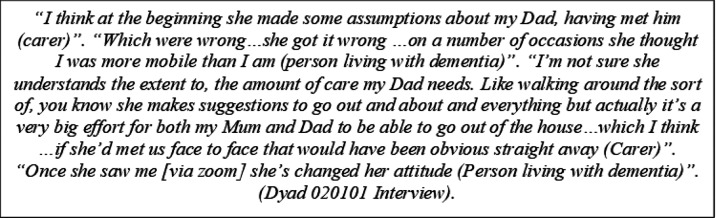


However, in light of the other observations made in this section, it could also be proposed that such suggestions were also made because the person with dementia and carer provided limited verbal information about their situation. This proposal can arguably be supported by their report that the DSW only changed their approach after they saw the person with dementia – not because of anything they said. This account highlights how remote engagement may influence the extent to which DSWs may need to work, interactionally, to ensure they have accurate understandings of need and so that they can subsequently make tailored, appropriate suggestions of support.

#### Theme Four: Carers’ ability to disclose privately

Although options to meet separately were provided, remote meetings between DSWs and people with dementia and carers (when recruited as a dyad) often involved the person with dementia and carer meeting with the DSW together. Opportunities to meet separately were often not taken up due to the carer not being able to leave the person with dementia on their own. DSWs reported concerns that this situation might prevent carers disclosing their true thoughts and feelings, for fear of upsetting the person they care for. In face-to-face interactions this issue was sometimes overcome by practitioner and/or carers making the most of an opportunity to speak without the person with dementia there (e.g., at the door, when the person with dementia visited the bathroom or when a cup of tea was being made). DSWs adapted to the reduction in these opportunistic moments when interacting remotely by offering carers the opportunity to engage via email. When making this offer, DSWs made it clear that what was emailed to them would be kept confidential, unless the carer thought it would be helpful for the topic to discussed with the person with dementia.


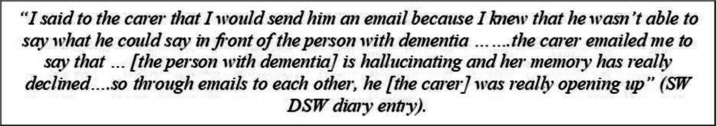


While we cannot know whether DSWs would have reported similar experiences if they had supported these people in face-to-face meetings, the reports showed that DSWs felt that the option to remotely interact via email aided carer disclosure about their current situation.


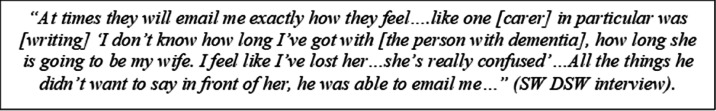


However, practitioners also voiced concerns about the use of email. One DSW stated she “*didn’t like*” how interacting solely with carers through email led to the “*carer [having] all the say*” (SW DSW diary) on whether the person with dementia wanted to be involved in her support. She felt unable to establish with the person with dementia that she was there for both of them. In addition, the SW DSW supervisor was concerned about the impact of writing and responding to emails on DSW workload.


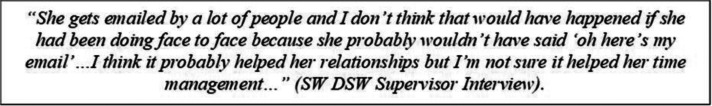


Carers also highlighted that the use of emails to aid communication would not be suitable for everyone, for example if they did not have access to a computer or had a learning difficulty or visual impairment.


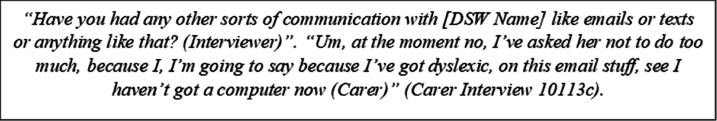


However, when there was a technological barrier, related to knowledge, DSWs addressed this by supporting people they worked with to learn how to use remote means for interacting (email/using video software); providing an opportunity for the person with dementia/carer to learn a new skill that could aid interactions with others, as well as them.


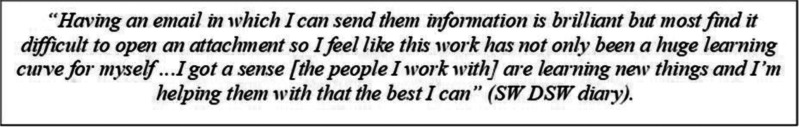


## Discussion

This study sought to examine experiences, perceptions and observations relating to whether D-PACT DSWs were able to remotely develop the type of understanding required to deliver PCSP. Findings suggest that DSWs’ ability to understand what matters most to the people they support within their current life situation, was impeded by working remotely. Specifically, the lack of environmental stimuli and physical proximity, peoples’ tendency to downplay or omit details of their difficulties within their talk, and carers’ reduced ability to disclose privately, made developing a holistic understanding challenging.

The development of a personal connection between a practitioner and a person they support and care for has previously been identified as a precursor for engagement and effective communication in dementia studies (Alsawy et al., 2017; Downs & Bowers, 2014) and other health care settings (e.g. Wolf et al., 2017). The ability to forge a personal connection remotely was reported as being negatively impacted by the person with dementia, carer and DSW not being able to engage in types of face-to-face ritualistic behaviours (e.g. tea being made and drunk together) that arguably reduce social anxieties about meeting someone new for the first time by ‘normalising’ the encounter and creating interactional openings (Goffman, 1952). This may be especially important when a “social border,” imposed by social stigmas and ingrained by professionals’ practices when working with clients/patients (Rowe, 1999) needs to be crossed (e.g. practitioner-patient) (Smith, 2011).

The observation that people tended to downplay or omit trouble talk (sometimes opting for what seemed like a habitual, generic no problem-oriented response to a question about how they were), can be potentially supported and further explained by studies on how people do complaints. Studies have shown that when making a complaint, individuals are aware that, by making a complaint, they are making themselves vulnerable to a negative evaluation by the recipient of the complaint, who may attend to “the propriety or fairness or justice or accuracy with which [they] have reported some (external) events, or [their]motives in doing so” (Drew, 1998, p. 295- 296). Consequently, they will deliberately shape their complaints in ways that will minimise a negative evaluation of themselves (Edwards, 2005). People with dementia and carers may be especially vigilant of other how may view them due to prior experience of stigma (Nguyen & Li, 2020) and/or concerns about what may happen if they are viewed as unable to cope (Stefan et al., 2018).

DSWs responded to these challenges by adapting their approaches. These adaptions included: using/switching to certain types of questioning, picking up on cues and using prompt sheets and email as an additional means of building understanding. The benefits of email as a means of enabling greater disclosure by carers about concerns was an important finding, especially as a recent systematic review reported that remotely delivered interventions seem to be less acceptable to unpaid carers of people with dementia, compared to treatment as usual, waiting list or attention control groups (Gonzalez-Fraile et al., 2021). Consequently, further use of this remote engagement strategy will be further explored in the D-PACT evaluation, whilst bearing in mind the importance of providing technological support and the potential unintended consequences of over-reliance on email. For example, the disengagement of the person with dementia and fostering a power imbalance between the carer and person with dementia, plus the increased burden on the support worker. While the value of using emails, as an additional means for disclosing information for people with dementia was not reported in the data, this option should also be made available to them and the value for them explored.

The use of prompt notes in remote support meeting will also continue to be observed during the evaluation of the project. While it is important for DSWs to feel ready and comfortable about initiating discussions with the people they support, there is the concern that over reliance on these prompts may result in DSWs unintentionally focusing on what they have observed and regarded to be important, rather than on what the people they support deem to be important. One strategy for addressing this within the intervention design and training has been to place more emphasis on the importance of co-setting agendas for support meetings, so that people with dementia and carers have the opportunity to say what they think it would be important to focus on in the meeting.

In addition to DSWs’ strategies for overcoming barriers to their understanding, people with dementia and carers also adapted and found a way to help practitioners understand what mattered to them - by sharing photos and objects of interest to them via the video screen. Similar findings were reported in a study on the use of videoconferencing for people living with aphasia – with people using photos and other props to help charity staff members get to know more about their passions (Neate, Kladouchou, Wilson and Shams (2021). Such findings emphasise the importance of practitioners spending time to get to know the person with dementia and carer, recognising that listening, showing genuine interest and engaging in conversations/activities led by people they support can be just as (or more) helpful than the use of practitioner-led questions to forge a personal connection and develop their understanding of what matters to the people they support and their situation, regardless of whether the interaction occurs remotely or in person.

The study found that while practitioners recognised the value of collaboratively (rather than independently) completing the pictorial intervention tools, they struggled to adapt to using them remotely. The value of using pictorial tools to collaboratively develop understanding of what matters to people with dementia, especially those with communication difficulties, has been reported elsewhere (Haroon et al, 2022). Further research is needed that can provide evidence informed communication strategies and resources (i.e., training and software) on how pictorial tools can specifically be used remotely (and in-person) by practitioners to collaboratively develop the type of understanding they need to deliver PCSP.

In the evaluation stage of D-PACT, DSWs will be given the option to be trained in Talking Mats, a well-researched pictorial communication tool that helps people with communities difficulties to think through their responses and share them in both remote and in-person interactions (e.g., Stans et al., 2019). Findings related to how this helped DSWs to build the type of understanding necessary for PCSP will be shared in subsequent publications.

Successful means of ascertaining people’s current situations (including potential for risk) were reported in this study. However, the concerns and uncertainty practitioners expressed about whether they had gathered the right/enough information indicates that further guidance on how practitioners can remotely assess risk for patients with complex needs would be valuable.

In sum, interacting via a video call, rather than phone, provided greater opportunities for D-PACT DSWs to develop their understanding of what mattered to the people they supported and their situation. This suggests that video calls should be promoted where possible. However, for people who can only meet over the phone or who choose to meet this way with their healthcare professionals, it will be important to plan and implement strategies for practitioners to develop the understanding required for delivering PSCP via this medium. Creative approaches and training on how person-centred communication practices can be employed during phone calls will be valuable.

### Limitations

While the development of understanding required for personalised care was a focal point during data collection and analyses, it was not the sole focus. Consequently, opportunities to further explore and examine emerging areas of interest may not have been fully realised. Findings primarily focused on whether and how D-PACT DSWs were able to build their understanding on *what areas* of their lives mattered to the people they supported, and less on whether/how they built understanding of what mattered to people *in terms of the management of their care* i.e., their care preferences (topic only slightly touched on in insights relating to whether the plan of action could be collaboratively completed remotely). A study primarily focused on the remote delivery of the support may have perhaps led to more comprehensive insights into whether and how dementia practitioners were able to develop the understanding required to fulfil the support planning aspect of PSCP and other aims and objectives of personalised care, e.g., shared decision making. However, this would have also required a shift in focus from practitioner understanding, to shared or mutual understanding. – where the person with dementia and carer also develops an understanding of what matters to them and what might improve their situation through interactions with the practitioner.

While the study had to collect data remotely due to covid-19 restrictions at the time of data collection, it is important to note this may have affected how much people with dementia engaged in the interview process as well.

Lastly, the participant cohort was not diverse (mainly white and British), limiting our ability to explore commonalities of experiences and perceptions across a diverse sample. Future research should also not only aim to explore experiences/perception of remote support from a more diverse sample, especially in relation to socioeconomic group and ethnicity, but also to examine how, when and for whom specific communication strategies, identified as supporting remote interaction, work.

It is clear that, alongside this study, other studies (e.g. Neate et al., 2021; Tuijt et al, 2021) on the remote delivery of support for people with dementia and other complex conditions are yielding important insights that are transferrable to other health and social care settings. Moving forward, it would be advantageous for practitioners delivering support remotely to be able to draw upon a synthesised evidence base on what has been shown to work remotely across service sectors and patient groups both generally - but also for specific interactional goals/activities relating to personalised care, such as the development of shared or mutual understanding. It would then be advantageous for future dementia studies to examine, within dementia support meetings, how, when and for whom identified communication practices, which support certain aspects/goals of personalised care work. Dementia practitioners could then reflexively utilise a wider, evidence-informed, communication strategies toolkit for supporting individuals remotely.

## Supplemental Material

Supplemental Material - Practitioners’ ability to remotely develop understanding for personalised care and support planning: a thematic analysis of multiple data sources from the feasibility phase of the Dementia Personalised Care Team (D-PACT) intervention Click here for additional data file. Supplemental Material for Practitioners’ ability to remotely develop understanding for personalised care and support planning: a thematic analysis of multiple data sources from the feasibility phase of the Dementia Personalised Care Team (D-PACT) intervention by Hannah Wheat, Sarah Griffiths, Alex Gude, Lauren Weston, Cath Quinn, Sarah Morgan-Trimmer, Tomasina M Oh, Crispin Musicha, Leanne Greene, Mike Clark, Sarah Rybczynska-Bunt and Richard Byng in Dementia

## Data Availability

Anonymised transcripts of interviews, observation notes and observations are available for sharing on request, subject to approval by the CI and Sponsor, under an appropriate data sharing agreement.

## ORCID iDs

Hannah Wheat https://orcid.org/0000-0003-3211-6254

Sarah Griffiths https://orcid.org/0000-0002-2652-3163

Alex Gude https://orcid.org/0000-0002-8575-8819

Lauren Weston https://orcid.org/0000-0001-8735-2292

Cath Quinn https://orcid.org/0000-0003-4644-4603

Sarah Morgan-Trimmer https://orcid.org/0000-0001-5226-9595

Tomasina M Oh https://orcid.org/0000-0003-4662-3193

Crispin Musicha https://orcid.org/0000-0003-0482-3218

Leanne Greene https://orcid.org/0000-0002-5383-4362

Mike Clark https://orcid.org/0000-0003-4964-5005

Sarah Rybczynska-Bunt https://orcid.org/0000-0002-4284-1081

Richard Byng https://orcid.org/0000-0001-7411-9467
