# Supplementary material for: Practitioners’ ability to remotely develop understanding for personalised care and support planning: a thematic analysis of multiple data sources from the feasibility phase of the Dementia Personalised Care Team (D-PACT) intervention
Source: Dementia (London). 2023 Jun 24;22(7):1461–86. doi: 10.1177/14713012231185281 (PMC10521162; doi:10.1177/14713012231185281)
Supplement: Supplemental Material - Practitioners’ ability to remotely develop understanding for personalised care and support planning: a thematic analysis of multiple data sources from the feasibility phase of the Dementia Personalised Care Team (D-PACT) intervention [file sj-pdf-1-dem-10.1177_14713012231185281.pdf]

APPENDIX A: The What matters to you tool

The 'What Matters to you' tool from D-PACT Practitioner Manual V4.0

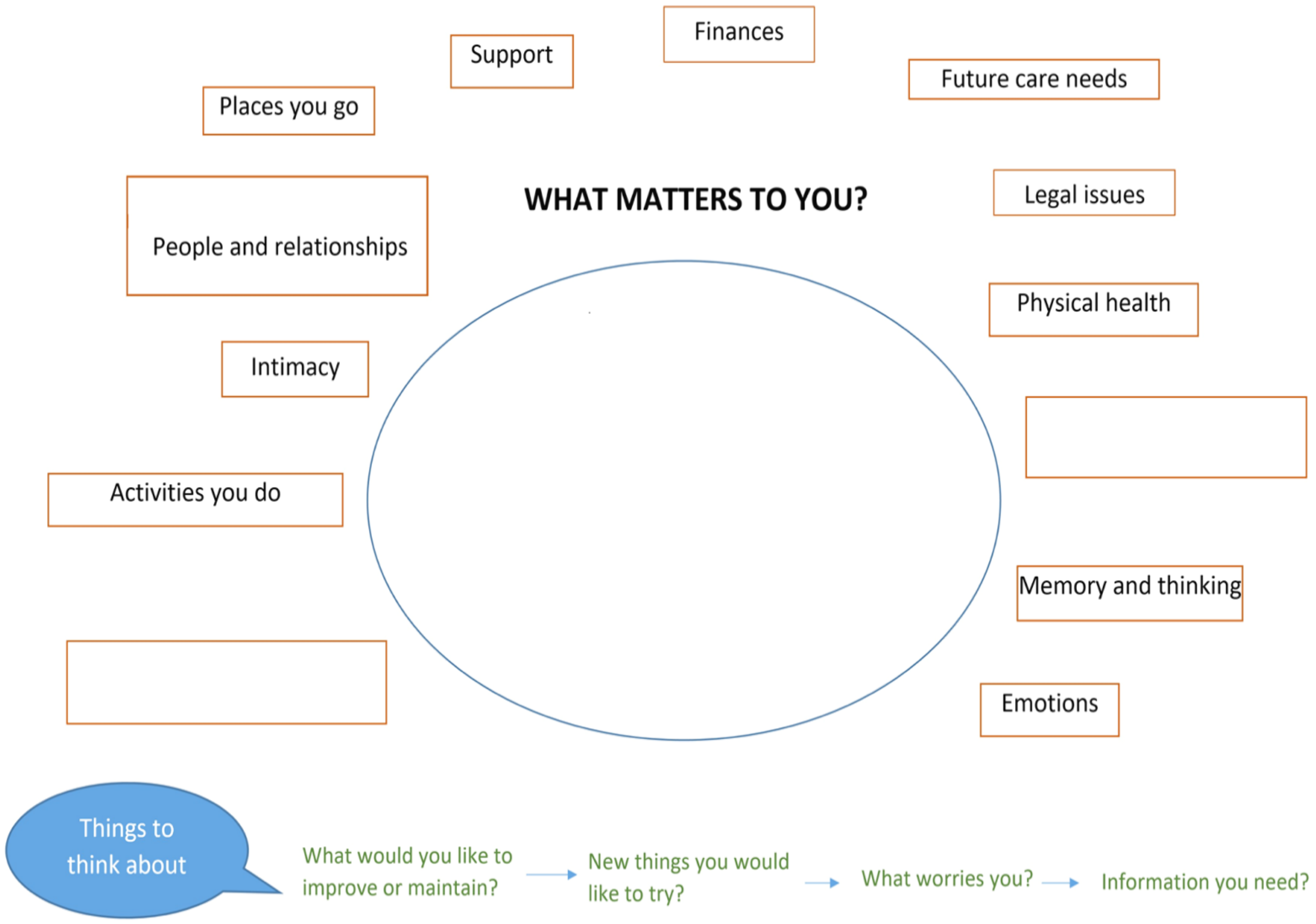

APPENIDX B: The Plan of Action

Plan of Action		
Who is the plan for?<br>Name of Dementia Support Worker:		
Action/s	Reason for action/s (how things are now)	Who will help this happen (people/organisations)? How?
When will we review this plan?		
